# Industrial Air Pollution Leads to Adverse Birth Outcomes: A Systematized Review of Different Exposure Metrics and Health Effects in Newborns

**DOI:** 10.3389/phrs.2022.1604775

**Published:** 2022-08-10

**Authors:** Triin Veber, Usha Dahal, Katrin Lang, Kati Orru, Hans Orru

**Affiliations:** ^1^ Institute of Family Medicine and Public Health, University of Tartu, Tartu, Estonia; ^2^ Institute of Social Science, University of Tartu, Tartu, Estonia; ^3^ Section of Sustainable Health, Umeå University, Umeå, Sweden

**Keywords:** air pollution, industrial air pollution, maternal exposure, adverse birth outcomes, ABO, low birth weight, preterm birth, small for gestational age

## Abstract

**Objectives:** To review the evidence of associations between adverse birth outcomes (ABO) and industrial air pollution.

**Methods:** Searches were conducted in PubMed, and Scopus databases, and additional articles were found from snowball search techniques. The included studies feature a study population of mothers with live-born babies exposed to industrial air pollutants, and they examine the effects of industrial pollutants on adverse birth outcomes—namely, low birth weight, term low birth weight, preterm birth, and small for gestational age.

**Results:** Altogether, 45 studies were included in this review. Exposure to PM_2.5_, PAHs, benzene, cadmium, and mixtures of industrial air pollutants and living near an industrial area affect birth outcomes.

**Conclusion:** This study concludes that industrial air pollution is an important risk factor for ABO, especially low birth weight and preterm birth. The strongest evidence is associations between ABO and air pollution from power plants and petrochemical industries. Understanding of specific chemicals that are critical to birth outcomes is still vague. However, the evidence is strongest for more specific air pollutants from the industry, such as PAH, benzene, BTEX, and cadmium.

## Introduction

The number of studies on the health impacts of industrial pollution is growing, yet reliable conclusions on health effects are still lacking [1]. One of the important health risks of maternal exposure to air pollution is adverse birth outcomes (ABO). The implications of ABO are not only on neonatal and childhood morbidity/mortality but have an effect over the life course [2, 3]. ABO has a causal relationship with chronic diseases such as coronary heart disease, hypertension, and non-insulin-dependent diabetes [2, 4], making it an important public health issue.

A growing body of systematic reviews, and meta-analysis recognizes ambient air pollution as an important risk factor for types of ABO like low birth weight (LBW), term low birth weight (TLBW), preterm birth (PTB), and being small for gestational age (SGA) [5–8]. The causes of ABO are not fully understood. The most common interpretations include systemic inflammation and alterations in the function of the autonomic nervous system [9–11]. Most studies have focused on some of the better-known general air pollution indicators, such as carbon monoxide (CO), nitrogen dioxide (NO_2_), fine particles (PM_2.5_), and particulate matter (PM_10_), as well as air pollution in general [7, 12–14]. However, the relationship of ABO with industrial air pollution and industry-specific air pollutants, such as benzene, polycyclic aromatic hydrocarbons (PAH), and heavy metals (e.g., cadmium and lead) is less studied. Thus, the evidence on the relationship between ABO and industrial air pollutants is still vague. As far as we know, there is no systematic review focused on industrial air pollution-related to ABO.

The objective of this paper is to review the evidence on how industrial outdoor air pollutants, including PM_2.5_, PM_10_, benzene, and BTEX (comprising benzene, toluene, ethylbenzene, and xylene), polycyclic aromatic hydrocarbons (PAHs), heavy metals, and mixtures of air pollutants, as well as industrial proximity, contribute to ABO—namely, PTB, LBW, SGA and term low birth weight (TLBW).

## Methods

### Inclusion Criteria and Search Strategy

The inclusion properties and the framing of the search terms and keywords were based on the PICO (Population, Intervention, Comparison, Outcomes) [15, 16] question: Do industrial outdoor air pollutants, including PM_2.5_, PM_10_, benzene, PAH, and heavy metals, contribute to ABO like PTB, LBW, TLBW, and SGA?

The criteria for including publications in the analysis were: 1) the study population consisted of mothers with a live-born child or children; 2) the mothers had been exposed to at least one industrial pollutant: PM_2.5_, PM_10_, PAH (including benzo(a)pyrene), benzene, BTEX, or heavy metals; 3) the study population was compared with mothers who were not exposed to industrial air pollution or whose exposure was significantly lower; and 4) the effects of industrial air pollution were studied for at least one of the listed birth indicators: PTB, LBW, TLBW, and SGA.

Publications with the following criteria were excluded from the analysis: 1) the effect of air pollution on birth outcomes was studied in the occupational environment or indoors, or 2) the study was not performed on humans (animal experiments). Conference summaries, pilot studies, and commentaries were also excluded.

The analysis included full-text, peer-reviewed scientific articles in English published before September 2020. The articles varied from clinical to epidemiological studies and included earlier review papers. The search was designed to find articles that deal specifically with the effects of industrial outdoor air pollution on PTB, LBW, TLBW, and SGA. PTB is defined as a delivery that occurs before 37 complete weeks of pregnancy [17]. LBW is defined as birth weight of less than 2500 g and TLBW as term delivery but birth weight <2500 g. SGA is defined as a weight below the 10th percentile for an infant born at a given gestational age [17].

We searched the PubMed and Scopus databases using the following terms: premature birth, preterm birth, birth effects, birth weight, small for gestational age, birth outcomes, gestation, industr*, petrochemical, plant, plants, metallurgical*, steel, polycyclic aromatic hydrocarbons, PAH, benzopyrene, bensopyrene, B (a) P, benzo (a) pyrene, benso (a) pyrene, fine particle, PM_2.5_, particulate matter, PM_10_, particles, benzene, and air pollution (Supplementary Material). In addition to using the PubMed and Scopus databases for the literature search, a snowballing search from the reference list of these publications was followed to find potentially relevant articles that were missed during the database search.

### Data Extraction and Analysis

We created a journal citation report and collected the following information from all the studies: study design, study area, number of observed births, exposure, outcome(s) assessed, and main result (Supplementary Table S1). The articles were saved and duplicates removed using Mendeley software. Then, the authors critically reviewed the included articles and conducted the quality assessment, in which one author completed the first round of review of all articles, and the other authors randomly selected reviewed articles and carried out a re-assessment. The process of inclusion, analysis, and interpretation of publications was carried out according to PRISMA (Preferred Reporting Items for Systematic Reviews and Meta-Analyses) guidelines [18].

## Results

### Search Results

**FIGURE 1:**
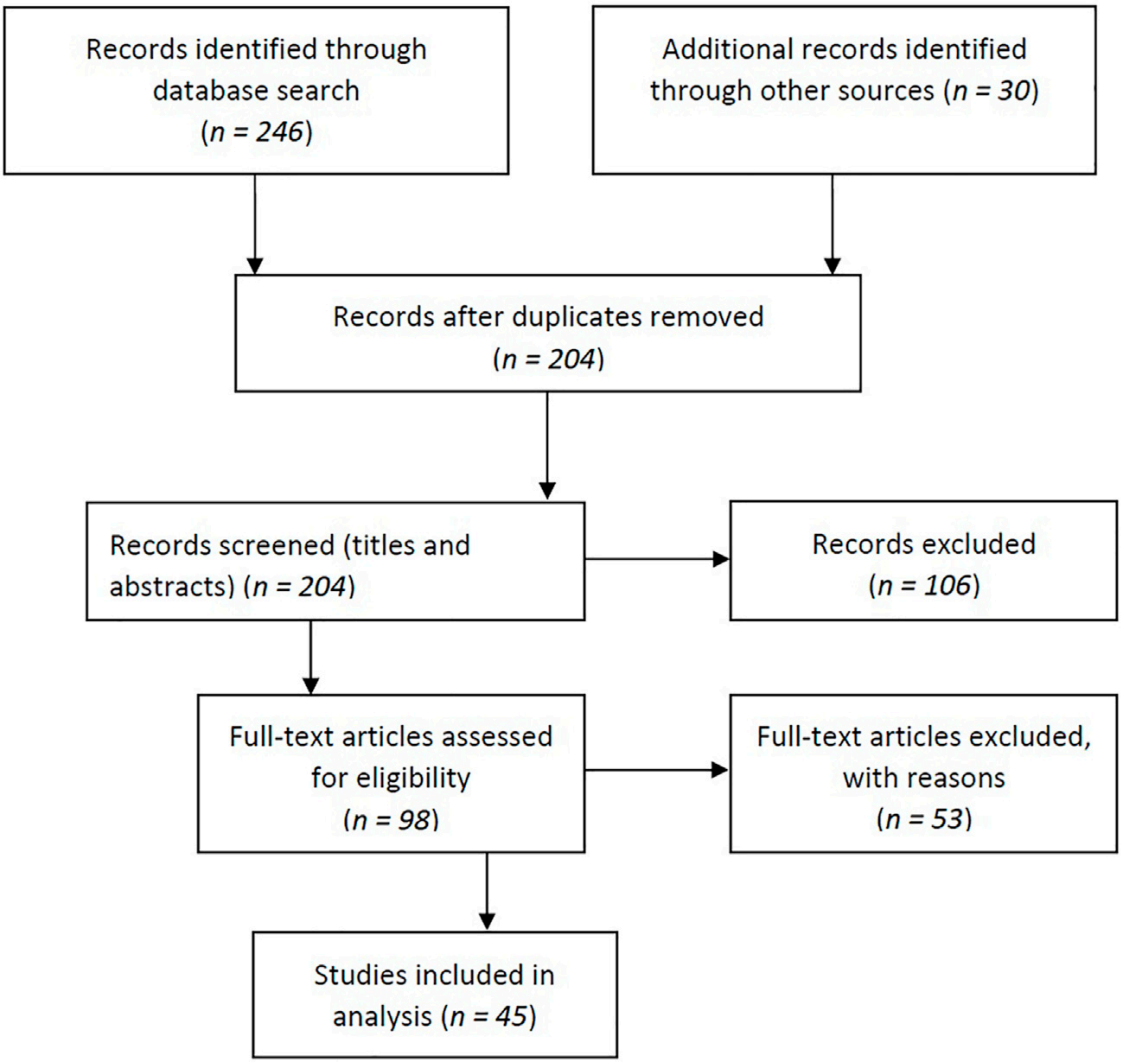
Flow diagram of article selection (Tartu, Estonia, 2022).

We found a total of 276 relevant publications, of which 161 publications were from Scopus, 85 were from the PubMed database, and 30 eligible articles were found using the snowball search technique. Subsequently, 72 duplicates were identified and removed. After screening the titles and abstracts, 106 articles were excluded. Based on the inclusion criteria, 98 full-text articles were subsequently assessed for eligibility. Of these, 53 articles did not meet all the criteria and were excluded. The main reasons for exclusion were: the effects were assessed indoors or in the occupational environment, the effects were assessed on animals, the full text was not available in English, or outcomes or exposure suitable for our study (i.e., the study covered air pollution in general, not specifically industrial air pollution) were not assessed. Finally, 45 articles were included in the review (Figure 1).

### Characteristics of the Eligible Studies

The major parameters from individual studies are summarized in the Supplementary Table S1. Among the 45 included articles, three of them are systematic reviews and reviews, and the rest of the articles are original studies. The included article dates from 1998, but most articles were published recently. To assess the relationship between air pollution and ABO, most articles included in the review used adjusted statistical models (usually a logistic or linear regression model) adjusted for other factors that could potentially affect ABO. Confounding variables varied from study to study, but in most cases, they adjusted for socioeconomic status, mother’s age, education, parity, and sex of the infant. Very often, the model was also adjusted for the season of birth, parents’ smoking status, nationality or race, and alcohol consumption. Studies concerning low birth weight were mostly adjusted for maternal weight, height and/or body mass index, and/or weight gain during pregnancy. Studies that focused on exposure to PAH, B(a)P, or benzene often measured cotinine levels in the mother’s blood to get a definite answer to the question of whether the mother smoked or not. Some studies also adjusted for the mother’s exposure to possible pollutants in food or at work, the use of vitamins (including folic acid) during pregnancy, stress, type of birth, day of birth, marital status, and health problems during pregnancy, such as gestational hypertension and gestational diabetes.

### Pollutants and Related Health Effects

In the analysis, we divided the types of birth outcomes according to the pollutant or exposure metric: 1) fine particles and particulate matter, 2) polycyclic aromatic hydrocarbons, 3) benzene and BTEX, 4) heavy metals, 5) mixtures of multiple air pollutants and 6) spatial and temporal indicators. We found that the most often studied air pollutants are fine particles, particulate matter, and PAHs, respectively. However, most studies have used spatial and temporal indicators. This means that only a few studies have used the direct measures of exposure assessment. Given the extensive research on LBW and PTB, most indicators have shown the effects on these birth outcomes. TLBW is the least studied birth outcome among the four birth outcomes we looked for.

#### Fine Particles and Particulate Matter

Extensive research aggregated into different systematic reviews has shown that fine particles (PM_2.5_) from different sources are associated with a negative effect on birth outcomes [8]. Furthermore, even low levels of PM_2.5_ are shown to affect ABO [12]. Associations specifically with PM_2.5_ from industrial sources are less studied, but most of the studies [12, 19–25] found PM_2.5_ inducing ABO: LBW [12, 21–24], PTB [21–23, 25], SGA [12, 21, 25], TLBW [12, 25], and IUGR [19, 20]. However, Cassidy-Bushrow et al. [26] found no association between PM_2.5_ and PTB. Higher PM_2.5_ levels have been reported near power plants, and it has been associated with higher risks of PTB, LBW, and IUGR [19, 20, 22, 23]. PM_2.5_ association with LBW has also emerged in residential areas located close to petrochemical industries [24].

In a cohort study in Ontario, Canada association with PM_2.5_ was not significant, but a strong association was found with SO_2_ [27]. The odds of women having an infant with LBW and PTB were 3.4 and 2.0 for a one-unit increase in SO_2_ exposure in µg/m^3^, where most of the emissions came from smelters and utilities*.* These results suggest that SO_2_ can be an important factor associated with PM_2.5_ in industrial air pollution affecting ABO. Yang et al. [28] also suggested that SO_2_ could be a precursor to ambient PM2.5 concentrations in their study region downwind of a coal-fired powerplant as mothers living as far as 20 to 30 miles downwind from a power plant during pregnancy had a higher likelihood of LBW and VLBW.

Majority of the studies [12, 21, 25, 26, 29–32] with particulate matter (PM_10_) from industrial sources have shown associations with ABO: PTB [12, 21, 25, 26, 32], LBW [12, 30–32], SGA [21, 25, 29], TLBW and [21, 25]. A case-control study conducted in Georgia, US, found higher odds of PTB with very LBW (<1500 g) in relation to maternal exposure to PM10 in counties with an industrial point source (OR = 4.31; 95% CI 1.88–9.87) [32]. A cohort study in the vicinity of a steel production complex, coke, cement, and lime plant in Brazil found the effect of PM_10_ and O_3_ on LBW [31].

However, there are some studies in which the effect of PM_10_ was not determined that are mainly related to waste incinerator exposures. For instance, no significant association between birth outcomes and PM_10_ near incineration plants has been found in the United Kingdom [33]. Also, in Italy, the association between high exposure to waste incinerator-induced PM_10_ and PTB was found only among primiparous mothers [34].

#### Polycyclic Aromatic Hydrocarbons

A majority of studies focusing on industrial air pollution showed an association between ABO and PAHs [19, 35–39]. This finding is consistent with an earlier systematic review [40] showing that PAHs as part of coal-fired power plant emissions are associated with LBW [35, 36], IUGR [19], and smaller head circumference (HC) [38, 39]. All studies included in this review that used B(a)P–DNA adducts as a PAH-exposure indicator [37–39] showed adverse effects of B(a)P on birth weight. Moreover, these studies have used cord blood testing as a good indicator of fetal metabolic condition at the time of delivery [41].

However, some studies did not find statistically significant associations [21, 29, 42]. Moreover, there is a discussion if PAH health effects could be related to particles. Two cross-sectional studies in the Czech Republic showed significant associations between IUGR and c-PAHs and not between IUGR and PM_2.5_ in the areas with high PAH concentrations but low concentrations of PM_2.5_ and PM_10_ [19, 20]. Dejmek et al. [19] suggested that the health effects of PM_2.5_ on ABO may actually be due to the effects of the PAHs attached to the fine particles. The same conclusion was reached by Jedrychowski et al. [43], who compared personally monitored prenatal pollutants PM_2.5_ and PAH, and found that the effect of PAHs were ten times higher than the effects of PM_2.5.,_ but in this study, the source of pollutants is unknown.

#### Benzene and BTEX

The vast majority of studies have shown negative effects from benzene on ABO. Two large-scale case-control studies conducted in Texas (United States) revealed the effect of benzene on birth weight [35, 36]. Among 78 studied chemicals, benzene was one of some that reduced birth weight [35], and among 449 studied chemicals, benzene was associated with the greatest incidence of reduced birth weight [36]. Two subsequent large-scale cross-sectional studies [21, 29] in Canada identified which industrial chemical emissions may affect birth outcomes. The first study [21], published in 2019 (birth events n = 2,525,645), showed a statistically significant association between benzene emissions and low birth weight. However, the latter study [29] (n = 32,836 infants) did not find a statistically significant association between critically ill small for gestational age (ciSGA) newborns and benzene.

The effects of BTEX on PTB were reported in a large-scale cross-sectional study based on 412,973 birth records in the United States [42]. A population exposed to increased coke facility emissions had 17% more premature births compared to a population exposed to lower emissions (below the median) (OR = 1.17; 95% CI 1.0–1.29). BTEX also increased the risk of PTB in a cohort study, where in the adjusted model, a 5 μg/m^3^ increase in BTEX concentration in the ambient air resulted in 1.54 (95% CI 1.25–1.89) times higher odds of PTB [26]. However, in a small study (107 pregnant women) in Thailand, no significant difference in the prevalence of LBW and SGA was found between mothers living in the petrochemical industrial area compared to those not living in the industrial area; nevertheless, the urinary metabolites of BTEX were higher among pregnant women living closer to petrochemical plants [44].

#### Heavy Metals

A biomonitoring study included in this review that measured the cadmium concentration in urine found an association between increased levels of cadmium and ABO [45]. This study, however, did not measure Cd concentrations in cord blood, which could reflect direct and more precise prenatal exposure. In a large-scale cross-sectional study of 61 different chemicals, the effect of cadmium, among other chemicals, was statistically significant [29]. However, in an epidemiological case-control study of 78 different chemicals, cadmium did not affect ABO [36]. Nevertheless, Govarts et al. [46] points out that chemicals that do not show significant associations at the single pollutant level can have stronger effects when analyzed as mixtures. They found that the association with birth weight was stronger when up to five chemicals (arsenic, lead, perfluorooctanoic acid-PFOA, Mono-(2-ethyl-5-carboxypentyl)phthalate-MECPP, and cadmium) were included in the analysis as a mixture [46].

The evidence of the effects of lead on ABO is inconsistent, but most cohort and case-control studies have shown an association between PLBW and LBW. The majority of the studies on lead included in this review found that increased lead exposure is related to ABO [29, 36, 42], but one found no association [35].

The evidence of ABO was often collected in areas with very high heavy metals like lead exposure levels. The residents of five towns in Shoshone County (US) were accidentally exposed to high levels of lead in air emissions during a 6-months period after a fire had damaged the pollution-control device of a local lead smelter plant in September 1973 [47]. Mothers exposed to high lead levels during that accident had a mean lead level in their blood of 164 mg/dl and had 2.4 times higher odds of having a baby with TLBW (OR = 2.4; 90% CI: 1.6–3.6) and 1.9 times higher odds to have a baby with SGA (OR = 1.9; 90% CI 1.3–2.8) compared to mothers who were not exposed to contamination in the control area [47]. However, the same study did not find any effect of lead contamination on PTB incidence.

#### Multiple Air Pollutants and Mixtures

In real life, industrial air emissions are composed of mixtures of multiple air pollutants, including those that we discussed above. Many of those are not routinely monitored and studied. Mixtures of different chemicals can have a different effect than the toxicity of individual chemicals because the higher dose intensity of the mixture or mixtures is more harmful than the individual pollutants alone [48, 49].

In Canada, 228 unique chemical emissions were analyzed primarily from energy (electricity and oil/gas) and mining-related sectors [21]. Twenty-four chemicals were identified, including ammonia, benzene, carbon monoxide, isopropyl alcohol, methyl ethyl ketone, styrene, and volatile organic compounds that affected SGA/TLBW/PTB. Another retrospective cross-sectional study identified hot spots, i.e., the metropolitan areas with the highest air pollution level based on the location of industrial facilities and prevailing wind trends, where 28 chemicals were identified that were associated with an increased risk of critically ill small for gestational age (ciSGA) infants [29].

In two case-control studies conducted in the US, the association between LBW and 78 and 449 different industrial chemicals, emissions were recognized. The first study identified 14 chemicals positively associated with LBW: benzene, benzo(g,h,i)perylenecumene, cyclohexane, dichloromethane, ethylbenzene, ethylene, mercury, naphthalene, n-hexane, propylene, styrene, toluene, zinc [35]. Second study identified five chemicals of which the highest odds were for exposure to acetamide (OR = 2.29; 95% CI 1.24–4, 20) and p-phenylenediamine (OR = 1.63; 95% CI 1.18–2.25) [36]. Significant risks were also found with exposures to 2,2-dichloro-1,1,1-trifluoroethane, tributyltin methacrylate and 1,1,1-trichloroethane [36]. In a study conducted in Canada, **a** higher risk for ABO was identified when mothers were exposed to industrial chemical emissions in mixtures of PM, CO, xylene, toluene, methylethylketone, 2-butoxyethanol, and n-butylalcohol [25].

#### Spatial and Temporal Indicators

The problems with unmeasured toxicants and effects of mixtures can be bypassed using spatial indicators (e.g., maternal residential proximity to industrial facilities) and temporal indicators (e.g., changes in the level of air pollutants, industry openings, and closings). We identified three systematic reviews on this topic [40, 50, 51]. Melody et al. [50] evaluated the effect of abrupt and major changes in outdoor air quality, including accidents like oil well fires and the suspension of industrial activities. During the 2008 Beijing Olympics (while much of the industry was shut down), PM_10_, NO_2,_ and SO_2_ levels in the air dropped significantly, leading to an average increase in birth weight by 23 g (95% CI 5–40 g) compared to infants born the following year [50]. Similarly, the closure of a Utah steel plant resulted in a lower risk of PTB (RR = 0.86; 95% CI: 0.75–0.98) compared to the plant’s operating period [50]. The tightening of power plant emissions regulations in the eastern United States reduced the prevalence of PTB and LBW [23].

Of the 25 original studies included in this review that used spatial or temporal indicators, 22 showed a negative impact on birth outcomes. Eight studies [21–23, 28, 52–55] showed that areas exposed to air pollution from power plants reported more ABO: LBW [22, 23, 28, 53, 55], PTB [21–23, 52, 54], SGA [21], and TLBW [21] compared to control areas or control periods. For instance, the odds of LBW (OR = 1.12, 95% CI: 1.03–1.22) or PTB (OR = 1.20, 95% CI: 1.14–1.25) were higher with the presence of more than one coal-fired power plant within a 20 km radius of a newborn’s home in the United States [22].

Birth outcomes were also negatively affected by the proximity of shale gas drilling [56], oil refinery plants [57, 58], petrochemical facilities [24, 30, 59–61], coke production, and steel-making facilities [42].

The evidence on proximity to waste incinerators is mixed, which could be due to the varied application of air pollution removal and control measures in waste incinerators [62]. Ghosh et al. [33] did not find effects on birth outcomes from the proximity of a waste incinerator. However, another study found an association between PM_10_ and PTB only for primiparous mothers when exposed to emissions from a waste incinerator [34]. Some other studies have also examined the associations of fireworks factories [63] and coke works [64] with PTB and LBW, respectively, but no associations appeared.

Living in an industrial area with different facilities also contributes to LBW and PTB [65–67]. Currie et al. [65] analyzed the impact of the opening and closing of 1,600 different industrial complexes on birth outcomes and property prices in the United States. The incidence of LBW increased on average by 3% within a mile radius per operating plant. Parker et al. [68] have reported that mothers who were pregnant around the time of the closure of the Utah Valley Steel Mill were less likely to deliver prematurely than mothers who were pregnant before or after. Moreover, preterm birth within the whole Utah Valley area did not change during the time of mill closure.

## Discussion

The present review looked for evidence between industrial air pollution, using different exposure metrics, and adverse birth outcomes. Subsequently we will discuss main limitation and strengths of the study and draw conclusion based on current knowledge.

### Limitations and Strengths

The present review may have excluded some eligible studies because we used only two databases to search for the relevant articles. Further studies could include more databases. Similarly, we assessed the quality of evidence-based on the study size and design. However, we have not specifically assessed the risk of bias in the included studies. Furthermore, the conclusions presented here can be affected by publication biases. Compared to articles with no confirmed relationships, articles that found pollution effects on ABO may be more likely to be submitted and published. Nevertheless, we have included only those articles with well-defined exposure of industrial sources, thus, the conclusion can be directly linked to the industrial sources of air pollution.

In our article search, we did not use search terms for all heavy metals, any kinds of mixtures, and spatial or temporal indicators, such as “lead,” “mercury,” “cadmium,” “proximity,” and “geographic location.” Thus, important industrial air pollutants (e.g., mercury) that can affect ABO are not included in this review. Nevertheless, articles related to some of these aspects emerged in our search results due to search terms like air pollution and industry* and from the snowball search. We decided to include the related articles in the review because they provide important industrial air pollution metrics, even though these topics were not covered by the systematic search.

To avoid double counting as described by Senn [69], we analyzed the reviews and original studies separately. In order to improve transparency, articles that are included in both the earlier systematic reviews and in this current review are marked with footnotes in the Supplementary Table S1.

### Conclusion

The current study has found strong evidence that industrial air pollution is an important risk factor for ABO, especially for LBW and PTB. The most robust associations with ABOs are with the air pollutants emitted from power plants and petrochemical industries. Many studies based on these industries have followed strong methodologies such as natural intervention studies (industry openings and closings), unique chemicals analysis, and PM measurements from industrial sources. However, no evidence of the negative effect of emissions from waste incineration plants was found.

Of the more specific air pollutants from industry, the evidence on ABO was more solid for the influence of PM_2.5_, PAH, benzene, BTEX, and cadmium. Most of the studies reviewed with PM_2.5_ from industrial sources have shown associations with LBW, PTB, SGA, TLBW, and IUGR. Some studies have suggested that the effect of PM_2.5_ can be enhanced through PAHs or heavy metals attached to particulates. The high lead exposure level is associated with LBW, but the association with PTB is unclear. The evidence reviewed here suggests that a mixture of multiple air pollutants has a stronger effect on ABO than single pollutants. Due to the difficulties in quantifying the concentration of several air pollutants, we found only a few studies that have counted large amounts of different chemicals emitted by industries and associated them with ABO. Thus, understanding the impact of specific chemicals and their dose is still vague.

Nevertheless, measuring more general spatial or temporal indicators often allows bypassing the problem of difficulties in measuring exact chemicals and concentration of air pollutants. For instance, we found strong evidence from natural intervention studies with spatial or temporal indicators on the association with ABO, especially LBW and PTB, which should be considered the most reliable as such studies also take into account the effects of multiple pollutant mixtures.

Regardless of the abundance of literature on associations between ambient air pollution and ABO, there is still high need for research focused on industrial sources of air pollutants. More studies with different methods in industrial exposure assessments are needed to clarify industrial effects on ABO. The evidence of long-term health effects of ABO is growing, however, more studies on several specific pollutants, their concentrations, and biological mechanisms as well as biomonitoring related to ABO are needed.

## Abbreviations

ABO, adverse birth outcomes; BTEX, benzene, toluene, ethylbenzene and xylene; CI, confidence interval; ciSGA, critically ill small for gestational age; HC, head circumference; IUGR, intra-uterine growth retardation; LBW, low birth weight; OR, odds ratio; PAHs, polycyclic aromatic hydrocarbons; PICO, population, intervention, control, outcomes; PLBW, preterm delivery of low birth weight; PM, particulate matter; PRISMA, preferred reporting items for systematic reviews and meta-analyses; PTB, preterm birth; RR, relative risk; SGA, small for gestational age; TLBW, term low birth weight.
