# Supplementary material for: Industrial Air Pollution Leads to Adverse Birth Outcomes: A Systematized Review of Different Exposure Metrics and Health Effects in Newborns
Source: Public Health Rev. 2022 Aug 10;43:1604775. doi: 10.3389/phrs.2022.1604775 (PMC9400407; doi:10.3389/phrs.2022.1604775)
Supplement: Supplementary file 1 [file DataSheet1.pdf]

Supplementary material

Industrial Air Pollution Leads to Adverse Birth Outcomes: A Systematized Review of Different Exposure Metrics and Health Effects in Newborns

Triin Veber, Usha Dahal, Katrin Lang, Kati Orru and Hans Orru

Scopus search:

(TITLE-ABS-KEY (industr*) OR TITLE-ABS-KEY (petrochemical) OR TITLE-ABS-KEY (plant) OR TITLE-ABS-KEY (plants) OR TITLE-ABS-KEY (metallurg*) OR TITLE-ABS-KEY (steel) AND TITLE-ABS-KEY ("polycyclic aromatic hydrocarbons") OR TITLE-ABS-KEY ("PAH") OR TITLE-ABS-KEY (bensopyrene) OR TITLE-ABS-KEY (benzopyrene) OR TITLE-ABS-KEY ("B(a)P") OR TITLE-ABS-KEY ("benso(a)pyrene") OR TITLE-ABS-KEY ("benzo(a)pyrene") OR TITLE-ABS-KEY ("fine particle") OR TITLE-ABS-KEY ("PM2.5") OR TITLE-ABS-KEY ("particulate matter") OR TITLE-ABS-KEY ("PM10") OR TITLE-ABS-KEY (particles) OR TITLE-ABS-KEY (benzene) OR TITLE-ABS-KEY ("air pollution") AND TITLE-ABS-KEY ("premature birth") OR TITLE-ABS-KEY ("preterm birth") OR TITLE-ABS-KEY ("birth effects") OR TITLE-ABS-KEY ("birth weight") OR TITLE-ABS-KEY ("small for gestational age") OR TITLE-ABS-KEY ("birth outcomes" [ ) OR TITLE-ABS-KEY (gestation)) AND (EXCLUDE (DOCTYPE , "cp") OR EXCLUDE (DOCTYPE , "ch") OR EXCLUDE (DOCTYPE , "no") OR EXCLUDE (DOCTYPE , "le") OR EXCLUDE (DOCTYPE , "ed") OR EXCLUDE (DOCTYPE , "sh")) AND (EXCLUDE (LANGUAGE , "Chinese") OR EXCLUDE (LANGUAGE , "French") OR EXCLUDE (LANGUAGE , "German") OR EXCLUDE (LANGUAGE , "Italian") OR EXCLUDE (LANGUAGE , "Spanish") OR EXCLUDE (LANGUAGE , "Polish") OR EXCLUDE (LANGUAGE , "Russian"))

PubMed Search:

((("premature birth"[Title/Abstract] OR "preterm birth"[Title/Abstract] OR "birth effects"[Title/Abstract] OR "birth weight" [Title/Abstract] OR "small for gestational age"[Title/Abstract] OR "birth outcomes"[Title/Abstract] OR "gestation"[Title/Abstract]) AND

(clinicaltrial[Filter] OR journalarticle[Filter] OR meta-analysis[Filter] OR randomizedcontrolledtrial[Filter] OR review[Filter] OR systematicreview[Filter])) OR (((("Premature Birth"[Mesh]) OR "Birth Weight"[Mesh]) OR "Infant, Premature"[Mesh]) OR "Gestational Age"[Mesh]) AND (clinicaltrial[Filter] OR journalarticle[Filter] OR meta-analysis[Filter] OR randomizedcontrolledtrial[Filter] OR review[Filter] OR systematicreview[Filter])) AND (((("industr*"[Title/Abstract] OR "petrochemical"[Title/Abstract] OR "plant"[Title/Abstract] OR "plants"[Title/Abstract] OR "metallurg*"[Title/Abstract] OR "steel"[Title/Abstract]) AND (clinicaltrial[Filter] OR journalarticle[Filter] OR meta-analysis[Filter] OR randomizedcontrolledtrial[Filter] OR review[Filter] OR systematicreview[Filter])) AND (((((("polycyclic aromatic hydrocarbons"[MeSH Terms] OR "polycyclic aromatic hydrocarbons"[tiab] OR PAH[tiab]) OR ("bensopyrene"[Title/Abstract] OR "benzopyrene"[Title/Abstract] OR "B(a)P"[Title/Abstract] OR "benso(a)pyrene"[Title/Abstract] OR "benzo(a)pyrene"[Title/Abstract])) OR ("fine particle"[Title/Abstract] OR "PM2.5"[Title/Abstract] OR "particulate matter"[Title/Abstract] OR "PM10"[Title/Abstract] OR "particles"[Title/Abstract] OR "Particulate Matter"[Mesh])) OR (("Benzene"[Mesh]) OR (Benzene[Title/Abstract]))) OR ("Air Pollution"[Mesh:NoExp]))) AND (clinicaltrial[Filter] OR journalarticle[Filter] OR meta-analysis[Filter] OR randomizedcontrolledtrial[Filter] OR review[Filter] OR systematicreview[Filter])) AND (clinicaltrial[Filter] OR journalarticle[Filter] OR meta-analysis[Filter] OR randomizedcontrolledtrial[Filter] OR review[Filter] OR systematicreview[Filter])) Filters: Clinical Trial, Journal Article, Meta-Analysis, Randomized Controlled Trial, Review, Systematic Review

Table S1. Characteristics of studies included in the analysis

Reference	Study area	Number of observed births	Exposure	Outcome(s) assessed	Main results
Systematic review and reviews					
Amster and Levy, 2019 [40][a]	N/A	N/A	$PM_{2.5}$, SO_2, PAH, proximity to industry	ABO[1], PTB[2], VPTB[3], LBW[4], VLBW[5], HC[6]	All four studies included in the systematic review on coal-fired power plants that measured the effects on birth outcomes showed adverse effects. $PM_{2.5}$, and SO_2 concentrations from coal plants were associated with ABO. The results were: higher odds of LBW, PTB, and VPTB among infants born within 20 km of more than one coal-fired plant; increased LBW and VLBW in <20 km of residential proximity; reduced birth head circumference and children`s birth weight when PAH-DNA adduct levels were above the median level; and post-closure of power plant decreased PAH-DNA adducts level and increased head circumference.
Marquès et al., 2020 [51][b]	N/A	N/A	proximity to industry	LBW, PTB, SGA[7]	The results clearly indicate that living near petrochemical complexes increases health risks. There is an increased risk of adverse effects on pregnancy and birth outcomes; basically, these were LBW, PTB, and SGA. The reported adverse effects are mainly related to exposure to $PM_{2.5}$ and PM_{10}, CO, NO_2, SO_2, O_3.
Melody et al., 2019 [50]	N/A	N/A	proximity to industry	PTB, BW[8]	There is some evidence that maternal exposure to acute changes in the air quality of short to medium-term duration increases the risk of fetal growth restriction and PTB. Oil well fires during the Gulf War in Iraq increased the PTB risk, but agricultural fires in Brazil did not. PM_{10}, NO_2, and SO_2 levels were lower during the 2008 Beijing Olympics due to industry closure, and the relationship with BW was shown. Infants born during eight months of the Olympic period were 23 g heavier than infants born to women pregnant in their eight-month in other years (95% CI[12] 5–40 g). The closure of a steel mill in Utah was associated with a transient improvement in air quality and reduced the likelihood of PTB (RR[13] = 0.86, 95% CI 0.75–0.98).

Reference	Study area	Number of observed births	Exposure	Outcome(s) assessed	Main results
Cohort studies					
Cassidy-Bushrow et al., 2020 [26]	Michigan, USA,	7,961	$PM_{2.5}$, PM_{10}, NO_2, BTEX	PTB	Prenatal benzene, ethylbenzene, toluene, and xylene exposure increased PTB. The results show that for every 5-unit increase in PM_{10}, there were 1.21 times higher odds of PTB (95% CI 1.07–1.38), and for every 5-unit increase in BTEX, there were 1.54 times higher odds of PTB (95% CI 1.25–1.89). However, no association between $PM_{2.5}$ or NO_2 was found with PTB.
Dolk et al., 2000 [64]	Great Britain	2,73,680	proximity to industry	LBW	No statistically significant association was observed between the LBW and residential proximity to coke works.
Ghosh et al., 2019 [26]	Great Britain	1,025,064 (births), 18,694 (infant deaths)	PM_{10}, proximity to industry	PTB, BW, SGA	There was no excess risk in relation to any of the outcomes: term BW, term SGA, PTB with either a mean modeled municipal waste incinerator (MWI) PM_{10} or proximity to the MWI.
Govarts et al., 2016 [46]	Flanders, Belgium	248	16 pollutants including Cd, Pb, As, mixtures of pollutants	BW	In single-pollutant models, arsenic was significantly associated with reduced BW. Chemicals not showing significant associations at a single-pollutant level contributed to stronger effects when analyzed as mixtures. The mixtures with the highest association with BW were composed of five chemicals, i.e., perfluorooctanoic acid (PFOA), lead, cadmium, arsenic, and Mono-(2-ethyl-5-carboxypentyl) phthalate (MECPP).
Ha et al., 2015 [22]	Florida, USA	423,719	$PM_{2.5}$, proximity to industry	PTB, VPTB, LBW	$PM_{2.5}$ exposure was significantly higher near coal and solid waste power plants compared to oil, gas, nuclear and other types of power plants. Infants born within 20 km of more than one coal-fired power plant had significantly higher odds of LBW (OR = 1.12, 95% CI 1.03–1.22), PTB (OR = 1.20, 95% CI 1.14–1.25), and VPTB (OR = 1.23, 95% CI 1.10–1.36).
Li et al., 2018 [63]	China	6059	proximity to industry	PTB	There was 3.6% PTB among the 6059 singleton live births. Density as a measure of exposure to fireworks factories was not significantly associated with spontaneous PTB or medically induced PTB. Data demonstrated that the residential density of fireworks factories appeared to be negatively correlated with the PTB rate.

Reference	Study area	Number of observed births	Exposure	Outcome(s) assessed	Main results
Reis et al., 2017 [31]	Brazil	12,541	PM_{10}, SO_2, O_3	LBW	The dose-response relationships verified LBW and concentrations of PM_{10} and O_3. An association between LBW and maternal exposure to SO_2 was not found.
Seabrook et al., 2019 [27]	Canada	25,263	$PM_{2.5}$, SO_2	PTB, LBW	Exposure to SO_2 was a top predictor of both LBW and PTB. Industrial sources of SO_2 account for 88% of the total emissions.
Tang et al., 2006 [38]	China	150	PAH (biomarker: benzo[a]pyrene (BaP))	BW, HC, BL[10]	High cord blood PAH-DNA adduct level (above the median of detectable adduct level) was associated with decreased birth head circumference and reduced infants/children's weight at 18, 24, and 30 months of age after controlling for potential confounders. A significant association was not found between birth weight and birth length. However, distance from the coal-fired power plant was not a good predictor for PAH exposure and birth outcomes.
Yang et al., 2002 [60]	Taiwan	39,750	Proximity to industry	PTB, TLBW	Residential exposure to air pollution specifically petrochemical industry pollution was not associated with TLBW and PTB.
Yang et al., 2002 [61]	Taiwan	57,127	Proximity to industry	PTB	The prevalence of delivery of PTB was significantly higher among mothers living near petrochemical industrial complexes compared to mothers living elsewhere in Taiwan. The OR was 1.18 (95% CI 1.04–1.34) for PTB in the petrochemically polluted region.
Yang et al., 2004 [58]	Taiwan	57,483	Proximity to industry	PTB	The prevalence of PTB was significantly higher in mothers living near oil refinery plants (OR: 1.14, 95% CI 1.01–1.28) than in mothers in control areas in Taiwan.
Yang et al., 2016 [45]	China	5364	Cd	PTB, LBW, SGA	Creatinine-corrected Cd level in maternal urine was associated with an increased likelihood of PTB for all infants (OR = 1.78, 95% CI 1.45–2.19) but was not associated with the likelihood of LBW (OR = 1.34, 95% CI 0.97–1.79) and SGA (OR = 1.05, 95% CI 0.88–1.26).
Yang et al., 2017 [28]	New Jersey, USA	1,676,798	$PM_{2.5}$, SO_2, proximity to industry	LBW, VLBW	Mothers living as far as 20 to 30 miles downwind from a coal-fired power plant during pregnancy have a higher likelihood of LBW and VLBW. The risk could increase by approximately 6.50% and 17.12% for LBW and VLBW, respectively. An increase of 1,000 tons of power plants' monthly SO_2 emissions during the last month of pregnancy increases the likelihood of LBW by 2.44%.

Reference	Study area	Number of observed births	Exposure	Outcome(s) assessed	Main results
Case-control studies					
Gong et al., 2018 [35]	Texas, USA	470,530	78 toxic chemicals, proximity to industry	LBW	Significantly higher odds of having LBW babies was associated with increased maternal residential exposure to benzene (OR = 1.06, 95% CI 1.04–1.08), benzo(g,h,i)perylene (OR = 1.04, 95% CI 1.02–1.07), mercury (OR = 1.04, 95% CI 1.02–1.07) styrene (OR = 1.06, 95% CI 1.04–1.08), toluene (OR = 1.05, 95% CI 1.03–1.07), and zinc (fume or dust) (OR = 1.10, 95% CI 1.06–1.13).
Gong et al., 2018 [36]	Texas, USA	470,530	449 toxic chemicals, proximity to industry	LBW	LBW could be associated with maternal residential proximity to industrial air emissions. The association was shown with five (toxic release inventory) TRI chemicals: acetamide, p-phenylenediamine, 2,2-dichloro-1,1,1-trifluoroethane, tributyltin methacrylate, and 1,1,1-trichloroethane. Association with other pollutants was also seen, for example, benzene (OR = 1.08, 95% CI 1.06–1.09), biphenyl (OR = 1.07, 95% CI 1.04–1.11), phenol (OR = 1.07, 95% 1.05–1.10), polycyclic aromatic compounds (OR = 1.06, 95% 1.04–1.08), mercury (OR = 1.05, 95% 1.02–1.08), lead (OR = 1.04, 95% 1.02–1.06), and lead compounds (OR: 1.03, 95% 1.01–1.04).
Rogers and Dunlop, 2006 [32]	Georgia, USA	325	PM_{10}, proximity to industry	PTB, VLBW	Maternal exposure to PM_{10} increased the odds of having PTB with VLBW compared to term delivery with normal BW in counties with an industrial point source.
Parker et al. 2008 [68]	Utah, USA	48,446	proximity to industry	PTB	Pregnant mothers around the time of Utah Valley Steel Mill Closure were less likely to have PTB babies, and the strongest effect was seen for exposure in the second trimester.
Cross-sectional studies					
Berkowitz et al., 2006 [47]	Idaho, USA	169,878	Pb	PTB, TLBW, SGA	During the high-exposure period, the exposed group had an increased prevalence of TLBW (OR = 2.4, 90% CI 1.6–3.6) and SGA (OR = 1.9, 90% CI 1.3–2.8) compared to the rest of Idaho. Increased risk for PTB in the exposed group was not found.

Reference	Study area	Number of observed births	Exposure	Outcome(s) assessed	Main results
Currie et al., 2015 [65]	Texas, New Jersey, Pennsylvania, Michigan, Florida, USA	152,282	proximity to industry	LBW	Incidence of LBW increased by roughly 3 percent within one mile of an operating toxic plant.
Dejmek et al., 1999 [20]	Teplice, Czech Republic	1943	$PM_{2.5}$, PM_{10},	IUGR[11]	Statistically significant associations between IUGR and exposures to high-level $PM_{2.5}$ (OR 2.11, 95% CI 1.20-3.70) and high (OR 2.64, 95% CI 1.48-4.71) and medium level PM_{10} (1.62, 95% CI 1.07-2.46) were found in the first month of pregnancy in a heavily polluted area with the chemical industry: surface mining, and large coal power plants.
Dejmek et al., 2000 [19]	Teplice and Prachatice Czech Republic	4854	$PM_{2.5}$, PM_{10}, PAH	IUGR	For each increase in 10 ng of a carcinogenic fraction of polycyclic aromatic hydrocarbons (c-PAHs) during the first gestational month (GM), IUGR was associated (OR = 1.22, 95% CI 1.07–1.39). Exposure to a high level of c-PAHs had an even higher risk of IUGR (OR: 2.15, 95% CI 1.27–3.63) compared to medium-level (OR = 1.60, 95% CI 1.06–2.15) exposure during first GM in the brown coal-exposed area.
Hansteen et al., 1998 [66]	Norway	3331	proximity to industry	BW	Significantly lower arithmetic mean BW was observed for newborns in the industrial residential area compared with the urban and rural area. Even controlling for gestational age, sex, parity, maternal smoking habits, and social class, residential location still had a significant effect on BW.
Lin et al., 2001 [57]	Taiwan		proximity to industry	PTB	The prevalence of PTB was significantly higher in mothers living in a petroleum refinery area (OR = 1.41, 95% CI 1.08–1.82) compared to controls in Taiwan.
Lin et al., 2001 [59]	Taiwan		proximity to industry	TLBW	TLBW was associated with proximity to the petrochemical municipality (OR = 1.77, 95% CI 1.00–3.12).
Lin et al., 2004 [30]	Taiwan	128,512	PM_{10}, SO_2, O_3, proximity to industry	LBW	LBW risk in an industrially polluted region (Kaohsiung) was 13% higher than in a non-industrial region (Taipei) (OR = 1.13, 95%, CI 1.03–1.24).
Mohorovic, 2004 [53]	Croatia	704	SO_2, proximity to industry	PTB, LBW	In the vicinity of coal power plants, greater and longer exposure to SO_2 emissions during the initial two months of pregnancy resulted in a significantly shorter gestation period and LBW.

Reference	Study area	Number of observed births	Exposure	Outcome(s) assessed	Main results
Nielsen et al., 2019 [21]	Canada	2,525,645	chemicals, proximity to industry	PTB, TLBW, SGA	Geographical differences in exposures and associations were observed in Canada. Among 228 studied chemicals, twenty-four chemicals were suspected to affect PTB, TLBW, and SGA.
Nielsen et al., 2020 [29]	Canada	32,836	chemicals, proximity to industry	critically ill SGA	The study estimated monthly wind dispersion of air emissions and calculated hot spots. 78 industrial chemical hot spots were associated with critically ill SGA hot spots. The highest positive association was seen with 28 chemicals, including particulate matter, heavy metals, volatile organic compounds, and carbon monoxide. No statistically significant relationship was observed with benzo(a)pyrene, PAH, and benzene. Cadmium, Lead, mercury, and biphenyl each had at least one significant association.
Phatrabuddha et al., 2013 [44]	Thailand	110	BTEX, proximity to industry	PTB, LBW	The urinary metabolites of BTEX were higher in pregnant women living closer to petrochemical plants. The pregnancy outcomes were not significantly different between the exposed and unexposed groups.
Porter et al., 2014 [42]	Alabama, USA	412,973	PAH, BTEX, Cd, Pb, As, Hg, proximity to industry	PTB, LBW	A significant association was found between PTB and residential proximity (≤5.0 km) to coke and steel production facilities. Metals (separately and as mixtures) and BTEX were significant determinants of PTB.
Serrano-Lomelin et al., 2019 [25]	Canada	333,247	mixtures of chemicals	ABO, PTB, SGA, TLBW	Pregnant women are at higher risk for ABO when exposed to chemical mixtures of PM, methyl-ethyl-ketone, xylene, carbon monoxide, 2-butoxyethanol, and n-butyl alcohol.
Perera et al., 2005 [37]	USA	170	B(a)P, BaP–DNA adducts, proximity to the World Trade Center	BW, SGA	The mean adduct levels in cord and maternal blood were highest among newborns and mothers who resided within 1 mile of the WTC site during the month after 11 September 2001. There were no independent fetal growth effects from either PAH–DNA adducts or environmental tobacco smoke, but adducts in combination with in utero exposure to environmental tobacco smoke were associated with decreased fetal growth.
Santoro et al., 2016 [34]	Italy	3153	PM_{10}, proximity to industry	PTB, LBW, SGA	The association between PM_{10} and PTB in the proximity of a waste incinerator was significant only for primiparous mothers. No significant results for the other investigated outcomes were observed.

Reference	Study area	Number of observed births	Exposure	Outcome(s) assessed	Main results
Ecological studies					
Hill, 2018 [56]	Pennsylvania, USA	1,098,884	proximity to industry	BW, LBW, SGA, PTB	The introduction of drilling increased LBW on average among mothers living within 2.5 km of a well compared to mothers living within 2.5 km of a permitted well. An additional well was associated with a 7% increase in LBW, a 5 g reduction in term BW, and a 3% increase in PTB.
Svechkina et al., 2018 [24]	Israel	7216	$PM_{2.5}$, SO_2, NO_X, proximity to industry	LBW	Increased LBW rate was associated with proximity to petrochemical facilities and with NOx and $PM_{2.5}$ exposure.
Tsai et al., 2003 [67]	Taiwan	64,215	proximity to industry	PTB	The prevalence of PTB was significantly higher among mothers living in the industrial area (OR = 1.11, 95% CI 1.02–1.21) compared to the control regions of Taiwan.
Tsai et al., 2004 [54]	Taiwan	23,072	proximity to industry	PTB	The prevalence of PTB was significantly higher among women living within 3 km of a thermal power plant (OR = 1.14, 95% CI 1.01–1.30) than among women living 3–4 km from a plant.
Intervention studies					
Casey et al., 2018 [52]	California	57,005	proximity to industry	PTB	Retirement of coal and oil power plants was associated with a decrease in the proportion of PTB within 5 km (-0.019, 95% CI -0.031, -0.008) and 5–10 km (-0.015, 95% CI -0.024, -0.007) from the plant while controlling for secular trends with mothers living 10–20 km away. For the proximity of 0–5 km, the decreased proportion indicates a reduction in preterm birth from 7.0% to 5.1%.
DeCicca and Malak, 2020 [23]	USA	25,892	$PM_{2.5}$, proximity to industry	PTB, BW	The reduction of power plant emissions, especially $PM_{2.5}$, after implementing the Clean Air Interstate Rule (CAIR) in the eastern USA reduced PTB and LBW. PTB was reduced particularly among those who were $\geq$ 35 years of age, had clinically-designated risky pregnancies, and had female newborns.
Yang and Chou, 2018 [55]	New Jersey, USA	150,623	SO_2, NO_X, proximity to industry	BW, PTB	The shutdown of the coal-fired power plant reduced the likelihood of having a LBW baby by 15% and the likelihood of PTB by 28% in New Jersey (located downwind from the power plant). After the shutdown of the

Reference	Study area	Number of observed births	Exposure	Outcome(s) assessed	Main results
					power plant, SO_2 emissions dropped by 99.99%, and NOx emissions also dropped by 99.95%.
Tang et al., 2014 [39]	China	308	PAH (PAH-DNA adduct levels)	BW, BL, HC	After the shutdown of a coal-fired power plant, PAH-DNA adduct levels reduced and it resulted in increased birth head circumference and increased weight at 18, 24, and 30 months.

[1]ABO – adverse birth outcomes, [2]PTB – preterm birth, [3]VPTB – very preterm birth, [4]LBW – low birth weight, [5]VLBW – very low birth weight, [6]HC – head circumference, [7]SGA – small for gestational age, [8]BW – birth weight, [9]TLBW – term low birth weight, [10]BL – birth length, [11]IUGR – intrauterine growth restriction, [12]CI – confidence interval, [13]RR – relative risk, [14]OR – odds ratio

[a]Following articles are included in this current review and in earlier systematic review by Amster and Levy, 2019:- Ha et al. 2015 (57), Yang et al. 2017 (60), Tang et al. 2006 (33), Tang et al. 2014 (36)

[b] [a]Following articles are included in this current review and in earlier systematic review by Marquès et al., 2020:- Lin et al. 2001 (63), Lin et al. 2001 (66), Yang et al. 2002a (68), Yang et al. 2002b (69), Tsai et al. 2003 (75), Svechkina et al. 2018 (67)
